# Catheter Fracture and Embolization Related to an Arm Venous Port

**DOI:** 10.1155/2011/763284

**Published:** 2011-10-05

**Authors:** Brent E. Burbridge

**Affiliations:** Medical Imaging, Royal University Hospital, 103 Hospital Drive, Saskatoon, SK, Canada S7N 0W8

## Abstract

This 55-year-old female had a chest X-ray during a follow-up visit for the management of her breast cancer. The chest X-ray demonstrated an embolized venous catheter superimposed upon the mediastinum. It was determined that the catheter of the patient's arm port had fractured and embolized to the pulmonary circulation. The catheter was retrieved, in the interventional radiology suite, under fluoroscopic guidance. The patient suffered no ill effects. Subsequently, one day later, the old vein port was removed and a new arm port and associated catheter were implanted to facilitate the delivery of the patient's ongoing chemotherapy.

## 1. Introduction

We commonly implant arm ports for patients who require long-term chemotherapy for malignancies. The port we employ most often is the Cook Vital Port, Mini Titanium (Cook Canada Inc., Stouffville, ON, Canada). It is a venous port system that is designed to have a small footprint and be utilized for intermittent venous access. This makes it an ideal device for long-term, intermittent, intravenous chemotherapy. In our experience, this device has been very reliable and demonstrates a low rate of complications [1]. Catheter fracture and embolization was not reported in a cohort of 125 patients with this device according to Burbridge et al. [1].

## 2. Case Report

This 55-year-old woman was diagnosed with breast carcinoma. The Cook Vital Port, Mini Titanium, had been implanted in her left arm for chemotherapy. The device was implanted subcutaneously and attached to the standard 5F catheter 664 days prior to extraction of the embolized catheter fragment. The device had functioned satisfactorily until this time, and the patient did not report any problems with the port prior to the chest X-ray that demonstrated embolization of the port catheter. The port had been accessed and flushed successfully, without difficulty, less than 30 days prior to the chest X-ray. The patient did not experience any arm trauma and did not report any unusual symptoms related to the left arm port.

**Figure 1:**
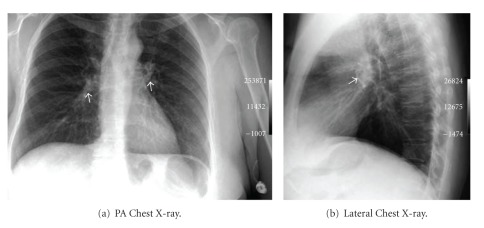
(a) The PA chest X-ray demonstrates the catheter fragment superimposed upon the mediastinum with portions of the catheter seen in both hilar regions (arrows). The arm port and the residual catheter are seen in the arm, cranial to the antecubital fossa, on the margin of the image. (b) The lateral chest X-ray demonstrates the catheter fragment superimposed upon the mediastinum (arrow).

The chest X-ray images (Figures 1(a) and 1(b)) demonstrated a small caliber catheter superimposed on the middle mediastinum with portions of the catheter in the expected location of the main pulmonary arteries bilaterally. The arm port and the residual catheter could be seen in the arm in the expected location proximal to the antecubital fossa, overlying the bicep muscle, on the PA chest image. It was obvious that the embolized catheter would need to be extracted.

Under fluoroscopic guidance, a 5F pigtail catheter (Cook Canada Inc., Stouffville, ON, Canada) was manipulated into the pulmonary arterial circulation via a right common femoral vein sheath. The pigtail catheter was manipulated into the left pulmonary artery using fluoroscopic guidance. The catheter was snagged with the pigtail and the catheter fragment and was pulled into the pelvic veins. Subsequently, a 2.5 cm Amplatz Goose Neck wire snare was deployed via a 6F catheter (EV3 Inc., Plymouth, MN, USA) and the catheter fragment was successfully extracted via the right femoral vein sheath. No complications were encountered during catheter fragment extraction.

**Figure 2:**
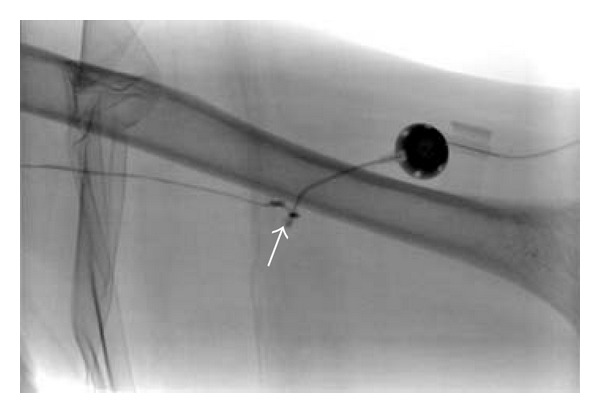
Contrast has been injected into the port. A small amount of contrast is seen to extravasate at the end of the residual catheter situated at the arm vein entry site (arrow). The contrast then flows freely into the patent, residual, fibrin sheath in the basilic vein related to the embolized catheter.

The patient returned to the department the next day to have her old port and residual catheter removed from her arm. Contrast was injected via the residual port, and it was noted that the contrast left the catheter stump and flowed down a patent fibrin tunnel in the patient's arm vein that had previously harbored the port catheter. In addition, there was a very small amount of extravasated contrast agent at the vein entry site. (Figure 2) The old port and residual catheter were removed, and a new Cook port was implanted to facilitate her ongoing chemotherapy.

## 3. Discussion

To our knowledge, Cook Vital Mini port catheter fracture in this location, associated with subsequent embolization of the catheter to the pulmonary arteries, has not been described in the literature previously. 

Weickhardt et al. reported catheter dehiscence in 2 of 92 patients (3.2%) related to detachment of the catheter at the port connection site. These two catheters embolized to the pulmonary circulation. In one patient of 92, the catheter did fracture at the vein entry site, but the catheter did not embolize to the pulmonary arteries. The ports in question, assessed by Weickhardt et al., were “Cook Interventional arm ports” [2].

Marcy et al. described catheter rupture, with contrast leakage at the vein entry site, related to a Bard arm port system, in 3/1,000 patients. However, there were no episodes of total catheter fracture and embolization in this patient cohort [3].

Surov et al. performed a systematic review of the literature between 1985 and 2007 and determined that during the timeframe assessed there were 143 reported incidents of catheter fracture and embolization related to subcutaneously implanted port systems. However, there was no mention of the anatomic location of the implanted ports and the manufacturers of the affected devices. In this cohort, it was found that a large number of catheter fractures were related to subclavian “pinch-off” syndrome consistent with chest implantation of the port system in question. No specific comments were made regarding arm placement of port systems in this systematic review [4]. 

Centrally implanted port systems are common and experience a variety of complications similar to arm implanted devices. Kock et al. amassed a cohort of 1,500 patients who received a subclavian vein-chest wall, port system. They reported an overall incidence of complications of 13%. Of this group, 2/1,500 (0.2%) experienced catheter fracture and embolization [5].

The cause for this complication is not readily evident in this instance. Presumably, it is related to wear and tear secondary to the prolonged implantation of this device. It may be related to the stresses of flushing and/or aspiration or due to catheter mobility at the vein entry site. Further investigation is warranted to attempt to determine the possible cause of this loss of catheter integrity.

## 4. Conclusion

This patient experienced an unexpected complication of her arm port. The embolization of the small caliber catheter was managed without adverse effect upon the patient. Surveillance of arm ports for this complication should consist of history, physical, and chest X-ray assessment to detect this rare problem. This complication can be treated with interventional radiology techniques.
